# Molecular docking between the RNA polymerase of the *Moniliophthora perniciosa* mitochondrial plasmid and Rifampicin produces a highly stable complex

**DOI:** 10.1186/1742-4682-10-15

**Published:** 2013-02-26

**Authors:** Bruno Andrade, Catiane Souza, Aristóteles Góes-Neto

**Affiliations:** 1 Departamento de Ciências Biológicas, Universidade Estadual do Sudoeste da Bahia, Jequié, Brazil; 2 Departamento de Ciências Biológicas, Universidade Estadual de Feira de Santana, Feira de Santana, Brazil

**Keywords:** *Moniliophthora perniciosa*, RNA polymerase, Rifampicin, Docking, MM/PBSA

## Abstract

**Background:**

*Moniliophthora perniciosa* (Stahel) Aime & Phillips-Mora is the causal agent of witches’ broom disease (WBD) in cacao (*Theobroma cacao*). When the mitochondrial genome of this fungus had been completely sequenced, an integrated linear-type plasmid that encodes viral-like RNA polymerases was found. The structure of this polymerase was previously constructed using a homology modeling approach.

**Methods:**

Using a virtual screening process, accessing the Kegg, PubChem and ZINC databases, we selected the eight most probable macrocyclic polymerase inhibitors to test against *M. perniciosa* RNA polymerase (RPO). AutoDock Vina was used to perform docking calculations for each molecule. This software returned affinity energy values for several ligand conformations. Subsequently, we used PyMOL 1.4 and Ligand Scout 3.1 to check the stereochemistry of chiral carbons, substructure, superstructure, number of rotatable bonds, number of rings, number of donor groups, and hydrogen bond receptors.

**Results:**

On the basis of this evidence we selected Rifampicin, a bacterial RNA polymerase inhibitor, and then AMBER 12 was used to simulate the behavior of the RPO-Rifampicin complex after a set of 5000 ps and up to 300 K in water. This calculation returned a graph of potential energy against simulation time and showed that the ligand remained inside the active site after the simulation was complete, with an average energy of -15 x 10^2^ Kcal/Mol.

**Conclusions:**

The results indicate that Rifampicin could be a good inhibitor for testing *in vitro* and *in vivo* against *M. perniciosa*.

## Background

*Moniliophthora perniciosa* (Stahel) Aime & Phillips-Mora is the causal agent of witches’ broom disease (WBD) in cacao (*Theobroma cacao*). When the mitochondrial genome (http://www.ncbi.nlm.nih.gov/genomes/GenomesGroup.cgi?taxid=4751&opt=organelle/) of this fungus had been completely sequenced, an integrated linear-type plasmid that encodes viral-like DNA and RNA polymerases was found [1].

RNA polymerase (RNAP) is responsible for transcribing DNA and is the direct or indirect target of most regulators of transcription [2]. The enzyme from the *M. perniciosa* mitochondrial plasmid (RPO) is a 766 amino acid DNA-dependent RNA polymerase belonging to the single chain family of polymerases, which occur in viruses and cellular organelles [3]. Its active site is distributed between two domains, Palm (Asp457 and Asp695) and Fingers (Tyr537 and Lys529), involved in transcription [3-5]. The mechanism of transcription by this enzyme shares several similarities with other multichain RNA polymerases [3,6], so it could have inhibitors in common with other polymerases.

Rifampicin is a macrocyclic molecule the ansamycin family. It contains a methyl-piperazinyliminomethyl side chain at position 3, a cyclopentyl-piperazinyliminomethyl side chain at position 3, and a cyclic spiro-piperidyl side chain at positions 3 and 4 [7]. This drug has been used since 1968 to combat *Mycobacterium tuberculosis* but is considered a broad-spectrum antibiotic [8,9]. It has a high capacity to bind and inhibit DNA-dependent RNA polymerases (RNAP) from bacteria through its specific interaction is with the polymerase β subunit [10]. The essential catalytic core of RNAP is evolutionarily conserved among all cellular organisms [9].

Virtual structure-based screening has become prominent in drug discovery, using protein targets [11,12]. Several free ligand databases are widely available today. Searching for molecules that can complex with target proteins can be done either by keywords (e.g. Kegg and PubChem databases) or by using a structure-activity relationship, available in the Zinc [13] and PubChem databases.

One of the most important techniques for receptor-based drug design is molecular docking [11]. Using crystallographic or modeled protein structures, molecular docking is often employed to screen compound libraries and to predict the conformation of a protein-ligand complex and calculate its affinity energy [12]. In general, docking programs such as AutoDock Vina [14] generate multiple protein-ligand conformations by sampling the ligand’s probable conformations in the binding pocket of the target protein, using flexible ligand-rigid receptor docking [11,14]. Scoring functions are used for docking calculations by these programs in an attempt to approximate the standard chemical potentials of the system [14]. AutoDock Vina uses a force-field-based scoring function approach to estimate binding affinities by calculating the non-bonded interactions based on traditional force fields, identify the correct binding position of a ligand, and rank ligands by their predicted binding affinities [11,14]. On the other hand, the problems of molecular docking as a screening tool have been widely discussed: the scoring functions are in general inaccurate and neglect solvent-related terms, and protein flexibility is ignored [12]. Coupled Molecular Docking and Molecular Dynamics is a good way to solve this problem because it can treat both proteins and ligands in a flexible manner, allowing the binding site to be relaxed around the ligand [11,12,15].

Molecular mechanics/Poisson-Boltzmann surface area (MM/PBSA) combines molecular mechanics energy and implicit solvation models and is more rigorous than most empirical or knowledge-based scoring functions [11,12]. It allows for rigorous free-energy decomposition into contributions originating from different groups of atoms or types of interaction [11,16]. In the MM/PB-SA method the free energy is calculated using snapshots of solute molecules obtained from explicit-solvent MD simulation [12].

The aim of this study was to search a series of molecules likely to form complexes with the RPO from *M. perniciosa*, available at the Kegg, PubChem and Zinc databases, and to select a potential inhibitor using a coupled Molecular Docking and Molecular Dynamics - MM / PBSA - approach.

## Methods

### Ligand searching

We searched initially by keywords and by nucleoside molecules in the Kegg (http://www.genome.jp/kegg/) and PubChem (http://pubchem.ncbi.nlm.nih.gov/) databases. Only molecules described as inhibitors of RNA polymerases were selected from the outputs. All 2D structures were copied in Similes format for comparison with other Zinc database (http://zinc.docking.org/) molecules; this increases the number of molecules that can be used for docking, starting from a known ligand. The 3D structures of these molecules were downloaded in mol2 and pdb formats for use in Virtual Screening, which was carried out by Molecular Docking and Molecular Dynamics. Following a protocol described by Irwin and Schoichet (2005) [13], molecules obtained from the Zinc database were selected for comparison, given 95-99% similarity, with the structures found in the Kegg and PubChem databases. In addition, selected structures were downloaded in mol2 and pdb formats for subsequent Docking Studies and Molecular Dynamics.

### Docking studies

The structures downloaded from the Kegg, Pubchem and Zinc databases were first checked in PyMOL 1.4 [17] to evaluate the presence or absence of hydrogen, the stereochemistry of chiral carbons, substructure, superstructure, number of rotatable bonds, number of rings and number of hydrogen acceptors and donors.

The ligand and receptor (RPO) molecules were prepared in AutoDockTools 1.5.6 [18]. All polar hydrogens were added to the receptor and Kollman United Atomic Charges were computed. For all ligands we added polar hydrogens and computed the Gasteiger charges. The grid definition, adjusted to the RPO active site, was set up manually by following the recommendations of the program manual [14,18]. The structures of the ligand and receptor were then saved in pqbqt format to be used for docking calculations. AutoDock Vina was used to perform Docking Scoring for each ligand-receptor complex [14]. Before running each Docking calculation, a configuration file was generated with information about grid size and coordinates and indicating the ligand and receptor files. The reports for each calculation were analyzed to obtain affinity energy (Kcal/mol) values for each ligand conformation in its respective complex. In addition, we used PyMOL 1.4 to verify the number of hydrogen bonds and non-covalent interactions between each ligand conformation and the catalytic residues of RPO that are involved in the recognition and polymerization mechanisms. In order to optimize the choice of an ideal complex we selected just one ligand that fit best in the RPO active site, considering all stereochemical aspects previously evaluated and the free-energy results.

### Molecular dynamics of complex

In this study we used the MM/PBSA protocol to calculate affinity and stability in the ligand-receptor RPO complex interaction, using the package Amber 12 [19]. Initially, we used the Antechamber program to make the ff99 force field recognize the types of atom in both ligand and receptor and avoid errors during the calculations.

tLEaP was used to neutralize charges (ff99 force field) and the RPO–ligand complex was immersed in a rectangular box of TIP3P water molecules. Following the protocol we used Sander to carry out a Molecular Dynamics (MD) equilibrium, restricted to a region of the protein that contains the active site (amino acids 457-695), according to the following parameters: 1000 cycles of steepest descent and 1000 cycles of conjugate gradient minimization, heating MD for 200 picoseconds (ps), density equilibrium for 200 ps, followed by Equilibrium Dynamics for 600 ps at constant pressure and 300 K. After the system equilibrated we followed this with the MM/PBSA protocol [11,19,20]. We then simulated a total of 4000 ps production steps of Molecular Dynamics, divided into four sets of 1000 ps (prod1, prod2, prod3 and prod4), saving the coordinates every 10 ps. Furthermore, we used the mm_pbsa.pl script to extract snapshots (without the water) from our production runs and obtain its trajectories. In addition, we checked the stability of the complex by plotting Potential Energy × Time (ps) and RMSD × Time (ps) graphs from all simulation trajectories. As a final step, we used the ambpdb command to generate a pdb file of the complex after the last stage of the Molecular Dynamics, and this structure was analyzed in PyMOL 1.4 to verify whether the ligand remained in the active site after the process was complete. In addition, Ligand Scout 3.1 [21] was used to generate 2D and 3D interaction maps of RPO-Rifampicin, presenting Hydrogen Bond Acceptors and Donors (HBA and HBD) and all hydrophobic interactions in the active site.

## Results and discussion

### Structures and binding energies of RPO complexes from AutoDock Vina

**Figure 1:**
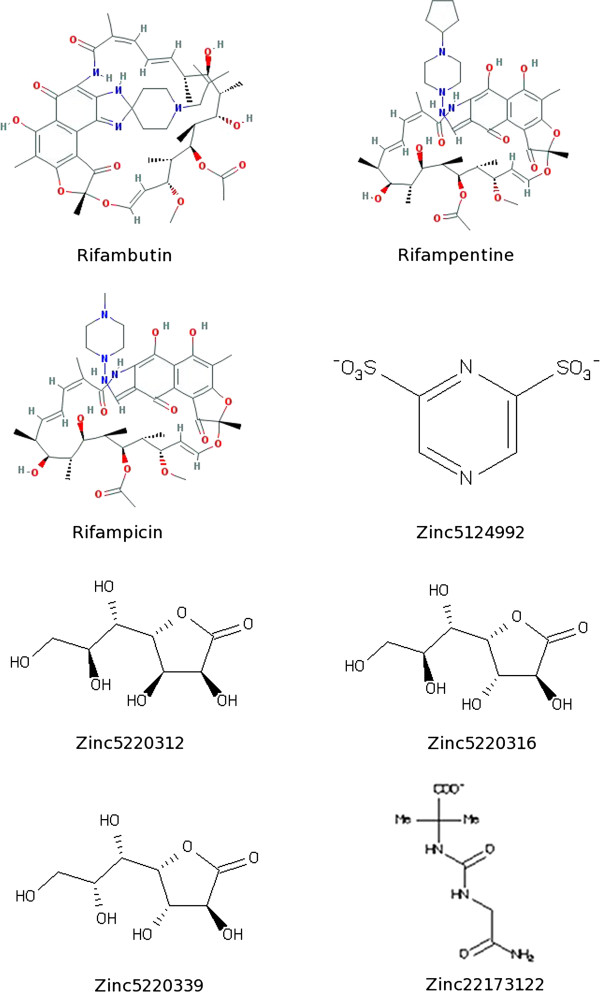
2D structures of the eight selected compounds used for docking studies.

**Figure 2:**
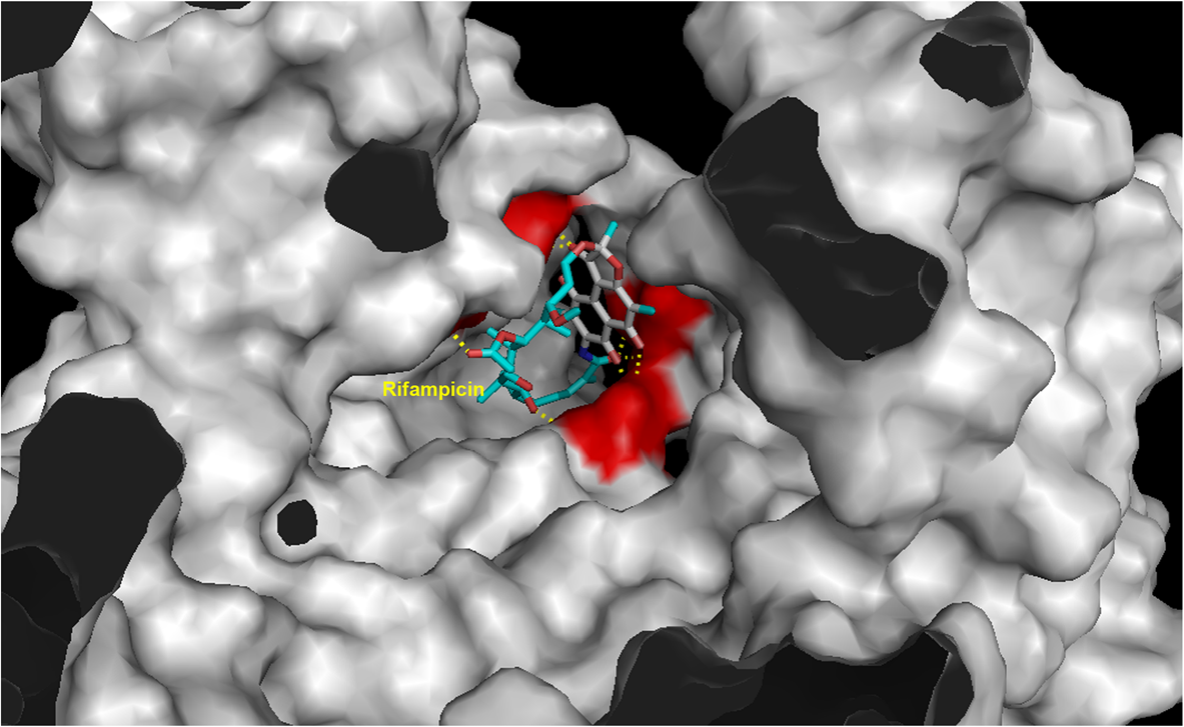
**RPO active pocket showing Rifampicin interaction.** The catalytic amino acids are in the red region.

**Figure 3:**
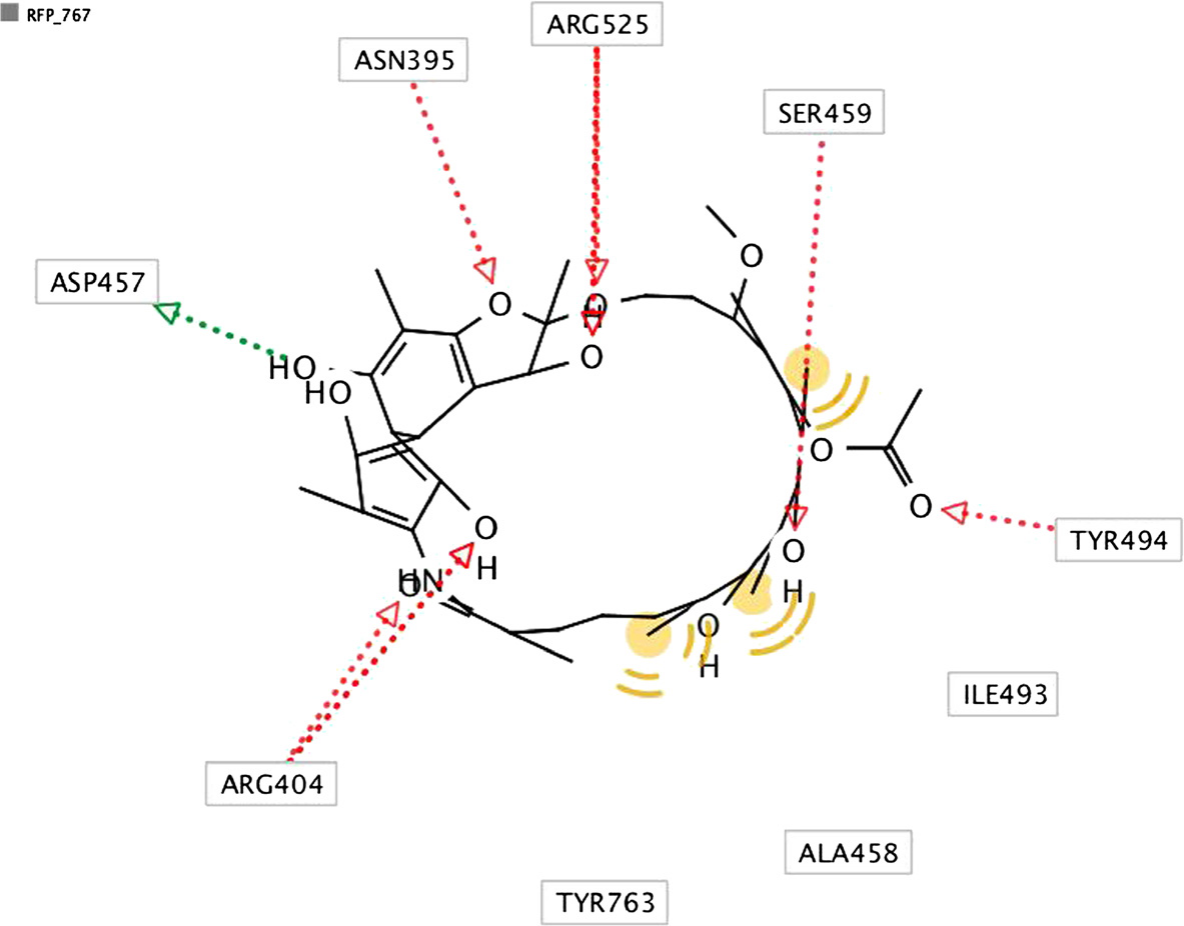
**2D interaction map presenting molecular interactions between RPO catalytic amino acids and Rifampicin.** Green arrow shows Hydrogen Bond Acceptor (HBA) and red arrow shows Hydrogen Bond Donor (HBD). Hydrophobic interactions are represented by yellow spheres.

**Figure 4:**
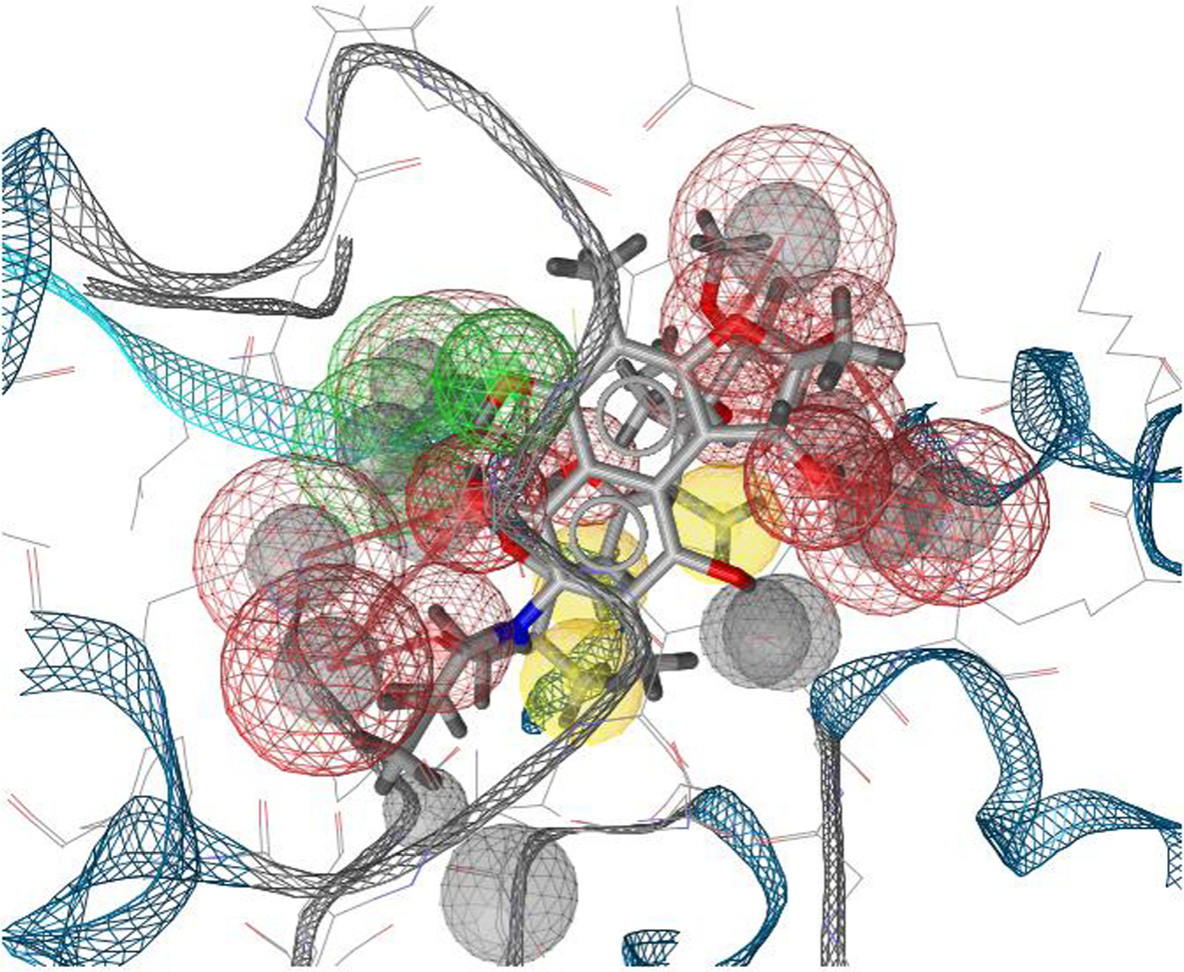
**3D interaction map presenting molecular interactions between RPO active site and Rifampicin.** Green spheres show Hydrogen Bond Acceptors (HBA) and red spheres show Hydrogen Bond Donors (HBD). Hydrophobic interactions are represented by yellow spheres.

After searching the KEGG, Pubchem and Zinc databases, we selected eight structures (Figure 1) that could interact strongly with RPO and belonged to different classes. Reliable prediction of complex interactions is essential for choosing a potential ligand in virtual screening methodologies, and that requires an appropriate tool capable of assessing the energy of the binding protein, indicating the quality of interaction [22]. The results of Molecular Docking with AutoDock Vina for different ligand-RPO complexes are presented in Table 1 in terms of the dominant configuration with highest affinity energy. The Docking scores returned by AutoDock Vina indicate that the ligand Rifampicin has the top rank as a good RPO inhibitor. We also evaluated other characteristics of all the ligands screened, such as H-bond donors and H-bond acceptors, and the capacity of at least one conformation of each ligand to bind to amino acids in the active site pocket of RPO when the complex is formed [3]. Among the molecules studied, Rifampicin bound best to the amino acids in the RPO active site and it presented a high affinity energy (Table 1) in docking calculations for all docking positions. In Figure 2 we show that Rifampicin fits inside the hydrophobic pocket of RPO. This molecule forms several hydrogen bonds (HBA and HBD) with amino acids in the RPO active site region - one with Asp457 (HBA), two with Arg404 (HBD), two with Arg525 (HBD), one with Ser459 (HBD), and two with Tyr494 (HBD) – as well as hydrophobic interactions with Tyr767, as can be seen in the molecular interaction maps (Figures 3 and 4). According to some authors, Asp457 in the active sites of the RPOs of several organisms is involved in transcription [3-5,23]. In *Escherichia coli* Rifampicin binds in a pocket of the RNAP β subunit deep within the DNA/RNA channel and blocks the RNA exit pathway [24]. In another study, Campbell et al. (2001) [9] described a 3.3 Ǻ crystal structure of *Thermus aquaticus* RNA polymerase complexed with Rifampicin, and the results of their biochemical experiments indicated that its predominant effect is to block the path of the elongating RNA transcript directly at the 5’ end when the transcript reaches either two or three nucleotides in length [25].

**Table 1:** Potential RPO inhibitors selected from the Kegg, PubChem and Zinc databases used in docking studies

**Molecule**	**Affinity (kcal/mol)**	**H-bond donor**	**H-bond acceptor**
Rifampicin	-10.4	6	15
Rifapentine	-9	6	15
Rifabutin Mycobutin	-8	5	14
Zinc5220312	-5.2	5	7
Zinc5124992	-5	2	8
Zinc5220316	-4.7	5	7
Zinc5220339	-5.1	5	7
Zinc22173122	-5.5	4	4

The use of RNA polymerases as molecular targets for virtual screening is not restricted to prokaryotes. An RNA-dependent RNA polymerase (RdRp) is an attractive target for anti-HCV agents [26]. However, we found no articles that dealt specifically with the use of inhibitors of fungal polymerases or polymerases encoded by mitochondrial genes. On the other hand, many authors report that all cellular RNA polymerases are relatively conserved in amino acid sequence and catalytic mechanism [2,7,9,23], We can therefore understand why we generally find the same class (macrocyclic) of RNA polymerase inhibitors acting on different groups of organisms. In addition, Rifampicin can probably act in *vitro* and *in vivo*, inhibiting mitochondrial transcription by RPO and thus blocking the mitochondrial metabolism of *M. perniciosa*.

### Molecular Dynamics MM/PBSA of RPO-Rifampicin complex

**Figure 5:**
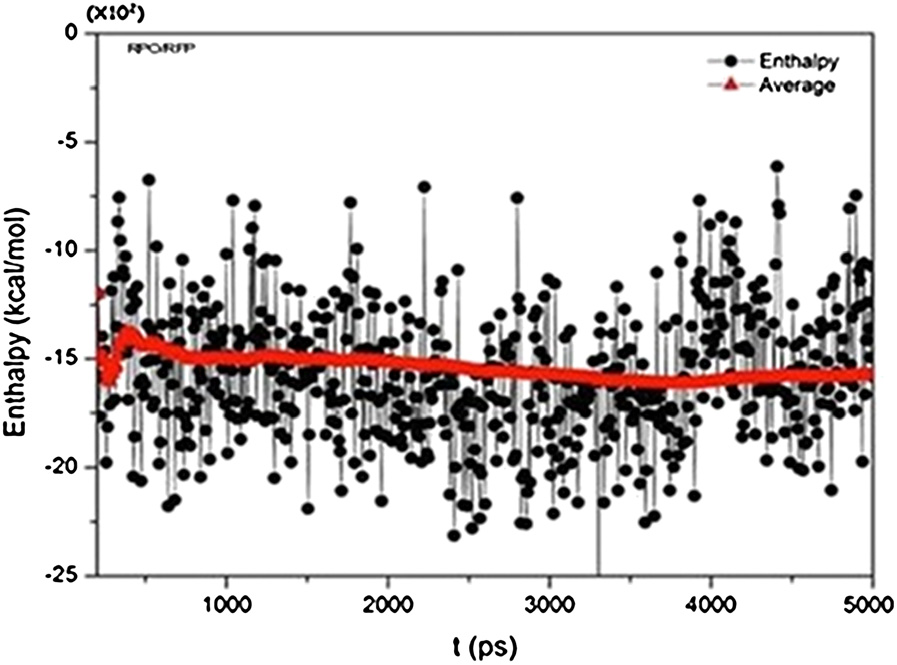
Graph of potential energy of RPO-Rifampicin complex during 5000 ps molecular dynamics simulation.

**Figure 6:**
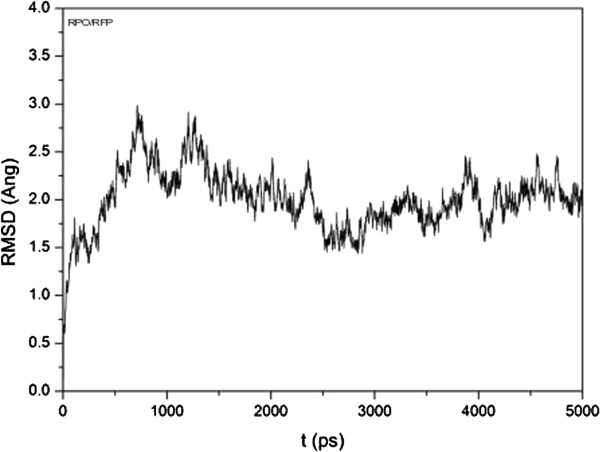
RMSD graph of RPO-Rifampicin complex during 5000 ps molecular dynamics simulation.

Using a Molecular Dynamics approach we analyzed the performance and stability of the RPO-Rifampicin complex. We then evaluated the potential energy of the complex during the simulation process and its final energy. The graph in Figure 5 shows that above 600 ps simulation the complex has already reached the average potential energy of the system, which is maintained until the end of the simulation. In addition, the potential energy during the plateau shows that the structure of this complex is perfectly plausible. The final energy reached at exactly 5000 ps was -15 × 10^2^ Kcal/Mol. We also considered the RMSD generated during all processes (Figure 6), and we noticed that the value converged around 1500 ps simulation.

The pdb of the complex, generated after molecular dynamics, showed that Rifampicin remains within the active site of RPO after 5000 ps of simulation. Furthermore, we can infer that this simulation time was sufficient to show that Rifampicin could block *M. perniciosa* RPO activity.

## Conclusions

In this article we describe the selection of one potential inhibitor of the *M. perniciosa* mitochondrial plasmid RNA polymerase from among eight molecules found in public databases, using a virtual screening approach. Rifampicin is a bacterial RNAPs inhibitor; nevertheless it forms a very stable complex with RPO, perhaps because this type of enzyme is highly conserved among organisms. Rifampicin forms complexes with exactly those amino acids in the active site pocket that are involved in transcription by RPO. This integration remained stable throughout the 5000 ps of Molecular Dynamics.

In a further step, we could analyze different mechanisms of Biomolecular Simulation to describe the mechanism by which Rifampicin inhibits the RPO, and whether Rifampicin will acquire different conformations within this process that can effectively interact within the active site of this enzyme.

We cannot discard use of the other ligands described in Table 1 as potential inhibitors of RPO or of others described as macrocyclic. Rifampicin was selected because it forms the best ligand-complex interaction. In future work we will perform biochemical tests *in vitro* and *in vivo* to verify whether our selected inhibitor can act effectively against *M. perniciosa* by making RPO-dependent transcription unfeasible.

## Competing interests

The authors declare that they have no competing interests.

## Authors’ contributions

BA carried out the ligand searching and Docking studies, performed the Molecular Dynamics of complexes, and drafted the manuscript. CS participated in the implementation of Molecular Dynamics and participated in its design and coordination. AGN participated in the design and coordination of this work. All authors read and approved the final manuscript.
